# Siloxane-spaced salen-type Schiff base cobalt complex. Experimental and docking analysis—a dual approach for evaluating anti-cancer efficacy

**DOI:** 10.1098/rsos.250279

**Published:** 2025-07-02

**Authors:** Madalin Damoc, Alexandru-Constantin Stoica, Mirela-Fernanda Zaltariov, Dragos Peptanariu, Mihaela Dascalu, Maria Cazacu

**Affiliations:** ^1^ Department of Inorganic Polymers, ‘Petru Poni’ Institute of Macromolecular Chemistry, Iasi, Romania; ^2^ Centre of Advanced Research in Bionanoconjugates and Biopolymers, ‘Petru Poni’ Institute of Macromolecular Chemistry, Iasi, Romania

**Keywords:** metal complex, Schiff base, anti-cancer activity, cobalt complexes, lipophilicity, molecular docking

## Abstract

A complex of Co(II), **CoL^1^**, with a salen-type Schiff base ligand, **H_2_L^1^**, having a siloxane spacer, was evaluated from the perspective of anti-cancer activity in comparison with a newly synthesized homologue under similar conditions, **CoL^2^**, but with a ligand with hexamethylene bridge, **H_2_L^2^**. Molecular docking simulations were used to estimate the possible interactions of the two cobalt complexes and their parent ligands with some key proteins involved in cancer development, the results indicating that the silicon derivatives are more potent anti-tumours. This is attributed to the conformational flexibility of the siloxane segment that favours establishing interactions with biological targets. Cytotoxicity assays against two cancer cell lines (MCF-7 and HeLa) also demonstrated significantly higher activity and selectivity for the siloxane-containing complex **CoL^1^,** compared with its fully organic ligand-based counterpart. The cytotoxicity of this complex on MCF-7 cell line showed a considerable effect at IC_50_ of 22.61 μM, compared with the one shown by **CoL^2^** of 43.82 μM. The dual experimental and theoretical approach provides valuable insights into the potential of designing Schiff base complexes with optimized therapeutic profiles and highlights the importance of the silicon structural motif in improving the efficacy of metal-based anti-tumour agents.

## 1. Introduction

The discovery of cisplatin triggered a major breakthrough in medicinal chemistry, mobilizing substantial efforts to explore metal-based drugs [1]. Despite its recognized efficacy against many types of cancer, the therapeutic use of cisplatin is hampered by severe side effects, limited selectivity and the emergence of cellular resistance to treatment. In response to these challenges, alternative research has focused on alternative metal complexes, such as Cu, Zn, Ti, Ni, Co, Ir, Ru, etc., which have demonstrated promising anti-cancer properties [2,3]. Among them, complexes of tetradentate Schiff bases such as salen and salophene have shown remarkable efficacy, surpassing cisplatin against various tumour cell lines such as MCF-7, HeLa and A431 (epidermoid carcinoma) [4,5]. Schiff bases themselves are a versatile class of organic compounds widely used for the development of pharmaceuticals and biologically active materials [6–9]. The discovery of the activity of Schiff bases and their complexes on various cancer cell lines has intensified research efforts to develop new drugs for this disease with minimal side effects [10–12]. Their easy synthesis, structural diversity and ability to form stable bonds with transition metals in various oxidation states have made them valuable in inorganic and bioinorganic chemistry. To date, at least 13 Schiff bases have entered clinical trials for cancer treatment and hypoxia imaging [13]. Their biological activities, including anti-oxidant, antimicrobial, antifungal, antiviral, anti-inflammatory, analgesic or anti-cancer properties, are often significantly enhanced when complexed with metals [10,14,15]. Salan Ti(IV) complexes, for example, have shown up to 30 times greater anti-cancer activity against colon cancer cells compared with cisplatin [16]. Also, cobalt, a trace element involved in essential biological processes such as haematopoiesis and amino acid and fatty acid metabolism, is emerging as a less toxic and more biologically compatible alternative to platinum [17], making cobalt-based complexes of interest for the development of new therapeutic agents [18,19]. A Co(III) Schiff base complex, Doxovir, has reached advanced clinical trials for its antiviral efficacy [20].

Despite these advantages, Schiff bases and their metal complexes have some drawbacks. They often suffer from limited stability in aqueous and/or acidic media, limiting their application to a narrow pH range (typically between 6 and 10) [21,22]. Their bioavailability is often suboptimal, negatively impacting their absorption and efficient distribution in biological systems. In addition, potential toxicity at therapeutic doses and a lack of specificity for biological targets may limit their clinical use [23–26]. Furthermore, some Schiff bases and their complexes are prone to rapid metabolic degradation, reducing their duration of action and therapeutic efficacy [27]. Addressing these challenges is essential to unlock the full pharmaceutical potential of these compounds.

Incorporation of a dimethylsiloxane containing segment into Schiff base structures is a promising strategy to alleviate these drawbacks [28,29]. The well-known conformational flexibility and hydrophobicity of silicone moieties create the prerequisites for an increase in the stability and bioavailability of Schiff bases, while reducing the potential toxicity [30,31]. The amphiphilic nature conferred by the combination of hydrophobic tetramethyldisiloxane and hydrophilic functionalized organic blocks facilitates self-assembly with formation of stable aggregates and adaptation to the polarity of their environment [32]. Consequently, this structural modification has the potential to improve the therapeutic efficacy and safety of Schiff-based drugs. In our previous work, we synthesized a series of salen-type Schiff bases with bis(propyl)tetramethyldisiloxane spacers and evaluated their electrical, magnetic, optical, catalytic and antimicrobial properties. Various coordination complexes with transition metals have also been developed and characterized [33,34].

Considering the unique properties of medicinal interest of cobalt complexes and their potential as chemotherapy agents [35–39], we explored the anti-tumour activity of our siloxane-bridged salen-type Schiff base Co(II) complex, for which the results of preliminary biological investigations have shown to be promising [34]. The study uses both molecular docking and experimental approaches to evaluate the cytotoxic efficacy. To highlight the effect of the siloxane bridge, a structurally analogous complex, but with a hexamethylene bridge, specifically synthesized and characterized for this purpose, was taken as a reference.

## 2. Experimental

### 2.1. Materials

**Scheme 1.**
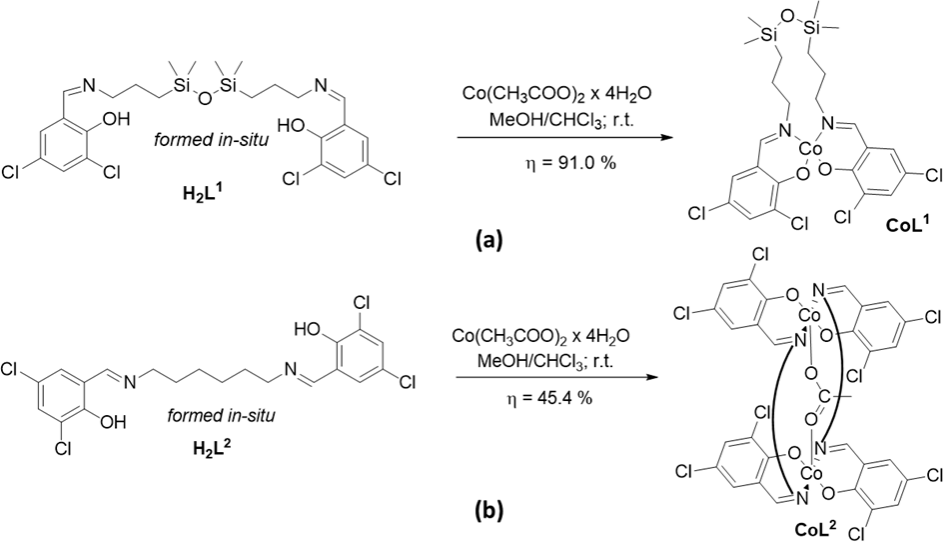
Reactions leading to the **CoL^1^** [34] (a) and **CoL^2^** (b) complexes of the *in situ* formed **H_2_L^1^** [40] and **H_2_L^2^** ligands (the brackets represent the hexylene spacer).

While the siloxane-spaced Schiff base, based on 1,3-bis(3-aminopropyl)tetramethyldisiloxane and 3,5-dichlorosalicylaldehyde, 6,6'-(((1,1,3,3-tetrametildisiloxan−1,3-diil)bis(propan−3,1-diil))bis(azanililiden))bis(metanililiden)bis(2,4-diclorofenol) (**H_2_L^1^**), could be synthesized and isolated according to the procedure described in [40], the derived cobalt complex, N,N'-[1,3-bis(propyl)tetramethyldisiloxane]bis-(3,5-dichlorosalicylideneiminato)-cobalt(II), **CoL^1^**, C_73_H_92_Cl_14_Co_3_N_6_O_9_Si_6_,CoL^1^·0.333CH_2_Cl_2_ was synthesized using a one-pot procedure (Scheme 1a), purified by crystallization from chloroform-methanol mixture, and fully characterized by elemental and spectral analysis as well as by single crystal X-ray diffraction (electronic supplementary material, figure S1) [34], which revealed that **CoL^1^** crystallizes in the triclinic (*P*−1) system. The asymmetric unit contains three distinct complex molecules and one co-crystallization solvent molecule, CH_2_Cl_2_. The compound is stabilized by π···π interactions between the aromatic rings, with a centroid-to-centroid distance of 3.6347(2)−3.3744(2) Å. MALDI-MS m/z: [M - H]^-^ theoretical for C_24_H_30_Cl_4_CoN_2_O_3_Si_2_ 647.9808; found, 647.4446 (electronic supplementary material, figure S2). Cobalt(II) acetate tetrahydrate, Co(CH_3_COO)_2_·4H_2_O, and solvents (chloroform and methanol) were purchased from Sigma Aldrich. Cell lines (HGF, human gingival fibroblast; MCF-7, human breast cancer and HeLa, cervical adenocarcinom) were obtained from Cell Lines Service GmbH (Eppelheim, Germany), Eagle’s minimal essential medium alpha (aMEM) and 1% penicillin–streptomycin–amphotericin B mixture (10K/10K/25 μg in 100 ml) from Lonza (Verviers, Begium), fetal bovine serum (FBS) from Biochrom (GmbH, Germany), Tryple from Gibco (Langley, VA, USA), phosphate buffered saline (PBS) from Invitrogen (Eugene, OR, USA) and CellTiter-Glo^®^ 2.0 Cell Viability Assay from Promega (Madison, WI, USA). Cisplatin (1 mg ml^−1^, concentrate for infusible solution) was achieved from Accord Healthcare.

### 2.2. Procedure

#### 2.2.1. Synthesis of reference compounds H_2_L^2^ and CoL^2^

The Schiff base derived from hexamethylenediamine and 3,5-dichlorosalicylaldehyde, 6,6'-(hexan−1,6-diilbis(azanililiden))bis(metanililiden)bis(2,4-diclorofenol), **H_2_L^2^**, was prepared according to the previously published procedure [40], and consisted of treating 0.05 g (0.26 mmol) 3,5-dichlorosalycilaldehyde dissolved in 5 ml methanol with a solution of 5 ml methanol with 0.015 g (0.13 mmol) hexamethylenediamine, stirring the mixture for 1 h at room temperature, followed by isolation by filtration of the yellow precipitate formed, and its washing with methanol and petroleum ether. The structure and purity of the product, **H_2_L^2^**, are according to [40]. Then, 90 mg of this product (0.19 mmol) was dissolved in 5 ml of chloroform and mixed with a solution of 0.048 g (0.19 mmol) of Co(CH_3_COO)_2_· 4H_2_O (0.19 mmol) in 5 ml of methanol, while stirring at room temperature for 1 h. The resulting mixture immediately precipitates giving an orange compound, **CoL^2^**. The latter was filtered, washed several times with fresh chloroform and dried in air. Crystals were grown from a suspension of the orange precipitate in dimethylformamide (DMF) and using di-*n*-dodecyl-dimethylammonium bromide as a surfactant. Yield: 44.5% (44.9 mg). IR (KBr pellet), υ_max_, cm^-1^: 1024 s (Si-O-Si), 1261 s (Si-CH_3_), 1616 s (CH = N), 2932 m, 2858 s (Si-CH_3_) (electronic supplementary material, figure S3). MALDI-MS m/z: calcd. for C_20_H_18_Cl_4_CoN_2_O_2_ + H^+^ [M+H]^+^, 519.95033; found, 519.81864 (electronic supplementary material, figure S4). Elemental analysis, calculated (%) for C_42_H_39_Cl_8_Co_2_N_4_O_6_ (Mw 1097,27): C 45.97, H 3.58, N 5.11; experimental: C 46.09; H 3.37, N 5.50.

### 2.3. Measurements

Fourier transform infrared (FTIR) spectra were recorded using a Bruker Vertex 70 FTIR spectrometer. The measurements were performed in the transmission mode in the range 400−4000 cm^−1^ at room temperature with a resolution of 2 cm^−1^ and an accumulation of 32 scans. Mass spectra were acquired on a BrukerRapiflex MALDI-TOF (BrukerDaltonics, Bremen, Germany) equipped with a Smartbeam three-dimensional laser. The samples (1 mg) were dissolved in chloroform and then diluted with a mixture of chloroform and methanol in the ratio of 3 : 2 volume. For the MALDI matrix solutions, 20 mg of α-cyano−4-hydroxycinnamic acid (HCCA) was dissolved in 1 ml methanol. Then, MALDI matrix solution and sample solution were mixed each other in 1 : 1, 2 : 1 and 5 : 1 ratios and finally 1 µl from each final solution was deposited onto the MALDI target, dried at room temperature and analysed in MALDI-TOF-MS. Mass calibration of MALDI-TOF-MS was performed by the peptide mixture standard solution (BrukerDaltonics, Bremen, Germany). FlexControl (BrukerDaltonics, v. 4.0) was used to optimize and acquire data using the following parameters: positive ion polarity in reflector mode, mass scan range (m/z 100−1600 Da), digitizer (1.25–2.5 GHz), detector voltage (1900–2117 V), 1000 shots per pixel and 10 kHz laser frequency. The laser power was set at 50 to 85% of the maximum and 1000 laser shots were accumulated for each spectrum.

### 2.4. Molecular docking calculation

The complexes were docked against the crystal structures of proteins (PDB ID: 1N8Z—resolution 2.52 Å [41], 2W3L—resolution 2.10 Å [42], 4GV1—resolution 1.49 Å [43], 4HJO— resolution 2.75 Å [44], 5DS3—resolution 2.60 Å [45], 5VAM—resolution 2.10 Å [44], 3CP9—resolution 2.50 Å [46], 5FDO—resolution 2.80 Å [47], 6GQO (resolution—1.87 Å) [48], 1FDW (resolution—2.70 Å) [49], 5GWK (resolution—3.15 Å) [50], 1P20 (resolution—1.34 Å) [49], 5O1H (resolution—1.32 Å) [50]), which were obtained from the Protein Data Bank (PDB) [51,52]. The GOLD (v. 2024.1) software suite was used to prepare the crystal structures for docking, i.e. the hydrogen atoms were added, water molecules and expedients deleted and the co-crystallized ligand identified: Human HER2 (1N8Z)—NAG (2-acetamido−2-deoxy-beta-D-glucopyranose); Chimaeric Bcl2-xL (2W3L)—DRO (1-(2-{[(3S)−3-(aminomethyl)−3,4-dihydroisoquinolin−2(1H)-yl]carbonyl}phenyl)−4-chloro−5-methyl-N,N-diphenyl−1H-pyrazole−3-carboxamide); PKB alpha (4GV1)—0XZ (4-amino-N-[(1S)−1-(4-chlorophenyl)−3-hydroxypropyl]−1-(7H-pyrrolo [2,3-d]pyrimidin−4-yl)piperidine−4-carboxamide); EGFR tyrosine kinase (4HJO)—AQ4 ([6,7-bis(2-methoxy-ethoxy)quinazoline−4-yl]-(3-ethynylphenyl)amine)); constitutively active PARP−1 (5DS3)—09L (4-(3-{[4-(cyclopropylcarbonyl)piperazin−1-yl]carbonyl}−4-fluorobenzyl)phthalazin−1(2H)-one); BRAF in Complex with RAF709 (5VAM)—92J (N-{2-methyl−5'-(morpholin−4-yl)−6'-[(oxan−4-yl)oxy] [3,3'-bipyridin]−5-yl}−3-(trifluoromethyl)benzamide); VEGFR2 kinase (3CP9)—C19 (3-(2-aminoquinazolin−6-yl)−1-(3,3-dimethylindolin−6-yl)−4-methylpyridin−2(1H)-one); Mcl−1 (5FDO)—5X2 (3-[3-(4-chloranyl−3,5-dimethyl-phenoxy)propyl]-{N}-(phenylsulfonyl)−1-{H}-indole−2-carboxamide); human KDR (VEGFR2) kinase (6GQO)—F82 (2-[4-(6,7-dimethoxyquinazolin−4-yl)oxy−2-methoxy-phenyl]-{N}-(1-propan−2-ylpyrazol−4-yl)ethanamide); Human 17-beta-hydroxysteroid-dehydrogenase type 1 mutant H221Q (1FDW)—EST (estradiol); Human topoisomerase II alpha (5GWK)—EVP ((5S,5aR,8aR,9R)−9-(4-hydroxy−3,5-dimethoxyphenyl)−8-oxo−5,5a,6,8,8a,9-hexahydrofuro[3',4':6,7]naphtho[2,3-d][1,3]dioxol−5-yl 4,6-O-[(1R)-ethylidene]-beta-D-glucopyranoside); DNA−5'-D(*CP*GP*AP*TP*CP*G)−3' (1P20)—DM2 (Doxorubicin); p53 cancer mutant Y220C (5O1H)—9GN (3-iodanyl−2-oxidanyl−5-propylsulfanyl−4-pyrrol−1-yl-benzoic acid). The scoring functions GoldScore (GS) [53], ChemScore (CS) [54,55], Piecewise Linear Potential (ChemPLP) [56] and Astex Statistical Potential (ASP) [57] were used with the GOLD (v. 2024.1) docking algorithm. The crystal structures of the **CoL^1^** and **CoL^2^** complexes were used for docking. For **H_2_L^1^** and **H_2_L^2^**, the X-ray structures were obtained from those of corresponding complexes by removing the metal, filling the free valences of the ligand with hydrogen atoms and optimizing the structure using the 6−31G basis set and the B3LYP method in the Gaussian16 software [58].

### 2.5. Cell culture and biological activity assessment

Biological activity was evaluated *in vitro* by CellTiter-Glo^®^ 2.0 Cell Viability Assay on three cell lines: MCF-7 as a model for breast cancer, HeLa as a model for cervical cancer and HGF (human gingival fibroblasts) as a model for healthy/normal cells. The cell lines were multiplied in tissue culture flasks with complete medium containing a mixture of alpha-MEM, 10% FBS and 1% penicillin–streptomycin–amphotericin B. The culture flasks were maintained at 37°C in a humidified environment with 5% CO_2_. Upon reaching confluency, the culture medium was removed and the cells were washed with PBS and then detached with Tryple.

To prepare for the assay, cells were seeded into 96-well white opaque plates at concentrations of 10 × 10³ cells/well for HeLa, 7 × 10³ cells/well for MCF-7 and 5 × 10³ cells/well for HGF, and then incubated overnight. The following day, the plates were treated with eight serial dilutions of the compounds (ranging from 3.9 to 500 μg ml^−1^) and cisplatin (ranging from 0.09 to 100 μg ml^−1^) to be tested. After 48 h, 100 µl of CellTiter-Glo reagent was added to each well according to the manufacturer’s protocol, and emitted light was measured after 15 min using a plate reader. Cell viability was calculated as the luminescence of the treated samples expressed as a percentage of the luminescence of the untreated cells. The experiments were repeated three times, and the results were analysed using GraphPad Prism software, v. 8.00 (GraphPad Software, San Diego, CA).

To compare the effect of **CoL^1^** and **CoL^2^** on the three cell lines (HGF, MCF-7 and HeLa), one way ANOVA test corrected with Dunnett’s test for multiple comparisons was used. To compare the effect of cisplatin on HGF and MCF-7 cell lines, paired two-tailed *t*‐test was used. Differences were considered statistically significant where the *p*-value was less than 0.05.

## 3. Results and discussions

### 3.1. Synthesis and structural analysis

In order to have a term of comparison for the biological activity of the **CoL^1^** complex (Scheme 1a), a homologous cobalt complex with an alkyl spacer was synthesized by the reaction with Co(CH_3_COO)_2_·4H_2_O of the Schiff base, **H_2_L^2^** (Scheme 1b), resulting from the condensation of 3,5- dichlorosalicylaldehyde with hexamethylenediamine in 2 : 1 molar ratio, in solution (methanol) at room temperature [40]. While the **H_2_L^2^** ligand was isolated in powder form and characterized as such, its cobalt complex **CoL^2^** was crystallized from DMF, using small amounts of surfactant (di-*n*-dodecyldimethylammonium bromide). The crystallographic analysis data obtained indicate the formation of a dinuclear neutral complex, in which the cobalt ions are interconnected, both by two deprotonated **H_2_L^2^** ligand molecules and by a bridging acetate ion, (μ-acetato-Co_2_(L^2^)_2_), coded still like **CoL^2^** (Scheme 1b, electronic supplementary material, figure S5). The chemical structure of this complex (**CoL^2^**) is supported by similar research papers from the literature, where the use of Schiff bases with poly(methylene) bridges leads to dinuclear metal complexes [59–61]. The oxidation state of the metal ions is mixed, respectively Co(II) and Co(III), and the coordination geometry of the cobalt ions is square pyramidal in *trans* configuration. From a supramolecular point of view, it forms a three-dimensional structure through C-H∙∙∙Cl interactions of 3.883(3) Å (electronic supplementary material, figure S6). However, given the low quality of the single crystals and analysis data collected, the structure of this mixed-valence dinuclear complex needs to be confirmed by other additional methods.

Mass spectra support the proposed structure by identifying the mass fragment m/z 519.81864 [**CoL**^**2**^]^+^ (theoretical, 519.95033) (electronic supplementary material, figure S4), while elemental analysis confirms phase purity. In the FTIR spectrum (electronic supplementary material, figure S3), the absorption band corresponding to the azomethine group, ν_CH=N_, is observed at 1616 cm^−1^, significantly shifted from 1647 cm^-1^ in the free ligand [40], indicating coordination to the metal ion.

### 3.2. Molecular docking

To theoretically estimate the anti-cancer activity of these compounds on the HeLa line, based on the literature [62–65], the following structures were selected from the PDB IDs: 1N8Z—resolution 2.52 Å [41], 2W3L—resolution 2.10 Å [42], 4GV1—resolution 1.49 Å [43], 4HJO—resolution 2.75 Å [44], 5DS3—resolution 2.60 Å [45], 5VAM—resolution 2.10 Å [45], 3CP9—resolution 2.50 Å [46], 5FDO—resolution 2.80 Å [47]. The two Schiff base ligands, **H_2_L^1^** and **H_2_L^2^**, and the corresponding metal complexes, **CoL^1^** and **CoL^2^**, were docked to the protein binding sites. The scoring functions GoldScore (GS) [53], ChemScore (CS) [54,55], Piecewise Linear Potential (ChemPLP) [56] and Astex Statistical Potential (ASP) [57] were used with the GOLD (v. 2024.1) docking algorithm. The GOLD docking algorithm is reported to be an excellent molecular modelling tool [66,67].

**Figure 1.**
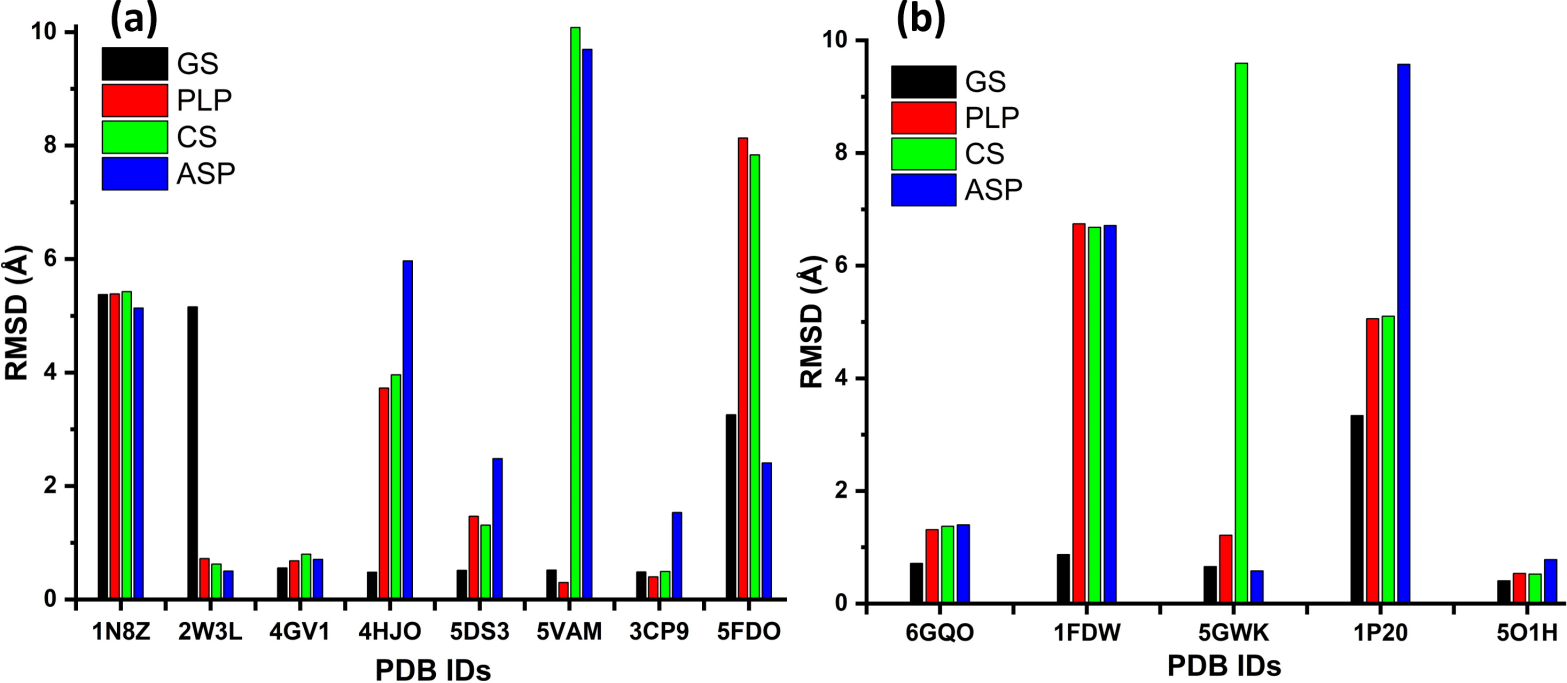
Comparative graphical representation of re-docking accuracy (expressed as root mean square distance, RMSD) for different proteins using the GS (GoldScore), PLP (Piecewise Linear Potential), CS (ChemScore) and ASP (Astex Statistical Potential) algorithms: (a) HeLa proteins; (b) MCF-7 proteins.

Re-docking accuracy, expressed as root mean square distance (RMSD) values for different proteins, estimated using the GS (GoldScore), PLP (Piecewise Linear Potential), CS (ChemScore) and ASP algorithms (electronic supplementary material, tables S1, S2), is presented in figure 1.

**Figure 2.**
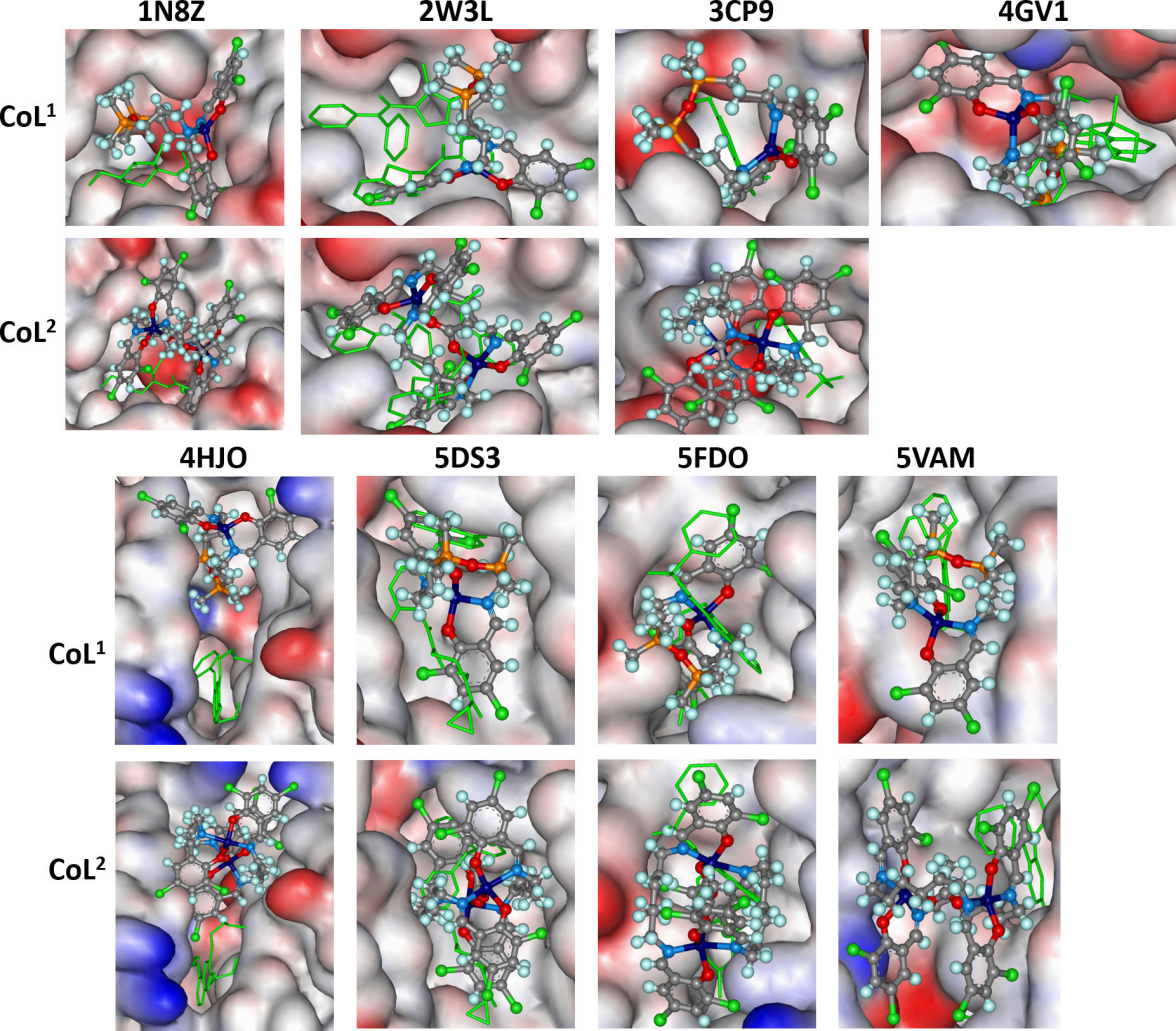
The docked poses of **CoL**^**1**^ and **CoL**^**2**^ (ball-and-stick) in different protein binding sites. The co-crystallized ligand is shown in line format (green), with hydrogens omitted for clarity. The protein surface is rendered, where blue indicates regions with a partial positive charge, red represents areas with a partial negative charge, and grey shows neutral regions.

The binding scores of the studied compounds in the protein pockets are graphically represented in figure 2, while the numerical values are given in electronic supplementary material, table S1.

For the **H_2_L^1^** and **H_2_L^2^** molecules, the docking modelling results indicate a favourable accommodation within the protein binding pockets (electronic supplementary material, figure S7). In all cases, a good overlap with the co-crystallized molecule was achieved, with calculated scores comparable to, and in some instances even better than, those of the co-crystallized ligand. The scores obtained for the two ligands are similar, although the **H_2_L^1^** molecule exhibits slightly higher values. This difference can be attributed to the highly flexible and hydrophobic bis(propyl)tetramethyldisiloxane moiety in **H_2_L^1^**, which provides significant conformational freedom, facilitating better accommodation within the protein pocket and promoting the establishment of hydrogen bonding and π···π interactions (electronic supplementary material, figure S8).

In all cases, the polar fragments, specifically the 3,5-dichlorosalicylic-substituted benzene rings, are positioned in the aqueous environment, which probably aids in better stabilization of the molecules. The Si-O-Si linkage in **H_2_L^1^** imparts a unique structural versatility, allowing the molecule to adopt various geometries, ranging from pseudo-linear to a *cisoid* configuration, where both aromatic rings are positioned on the same side. This structural flexibility contributes to a higher density of favourable interactions, such as stronger hydrogen bonding, which enhances the molecule’s fit within the binding pocket.

**Figure 3.**
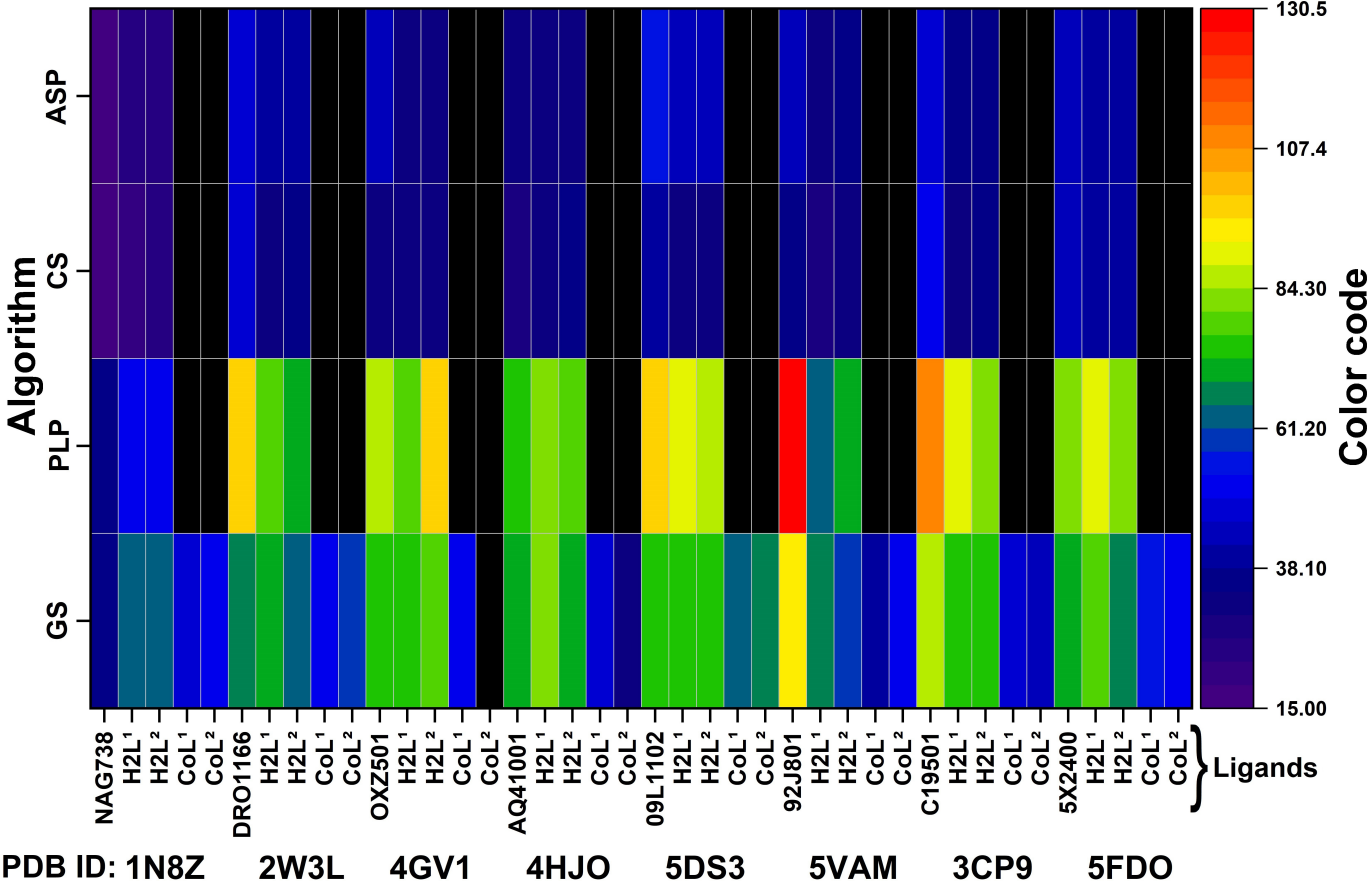
Heatmap representation of predicted binding affinities (depicted using a colour code) for specific proteins (PDB ID) in the HeLa cell line with different ligands, using the GS, PLP, CS and ASP algorithms.

Additionally, the pronounced lipophilic character of the bis(propyl)tetramethyldisiloxane segment in **H_2_L^1^**, compared with the hexamethylene group in **H_2_L^2^**, may further stabilize the ligand-protein complex. This stabilization occurs by securing the ligand within the hydrophobic pockets of the protein, thereby reducing the likelihood of water molecules from the exterior displacing the ligand. The increased flexibility of **H_2_L^1^** also facilitates the formation of several strong hydrogen bonds (e.g. -OH···O, -NH···O, -NH···N) and protein-azomethine hydrogen interactions, which are less prevalent in the case of **H_2_L^2^**. Regarding DNA interactions, the flexible nature of **H_2_L^1^** enables it to intercalate into the DNA helix and engage with nitrogenous bases through both aromatic rings. In contrast, the less flexible **H_2_L^2^** molecule is less capable of forming such interactions. The complexes **CoL^1^** and **CoL^2^** also show good scores indicating reasonable binding, similar to the co-crystalized ligand (figure 3, electronic supplementary material, table S1). Only GS runs for the metal complexes, because it is the only algorithm optimized for metal complex molecules figure 2.

**Figure 4.**
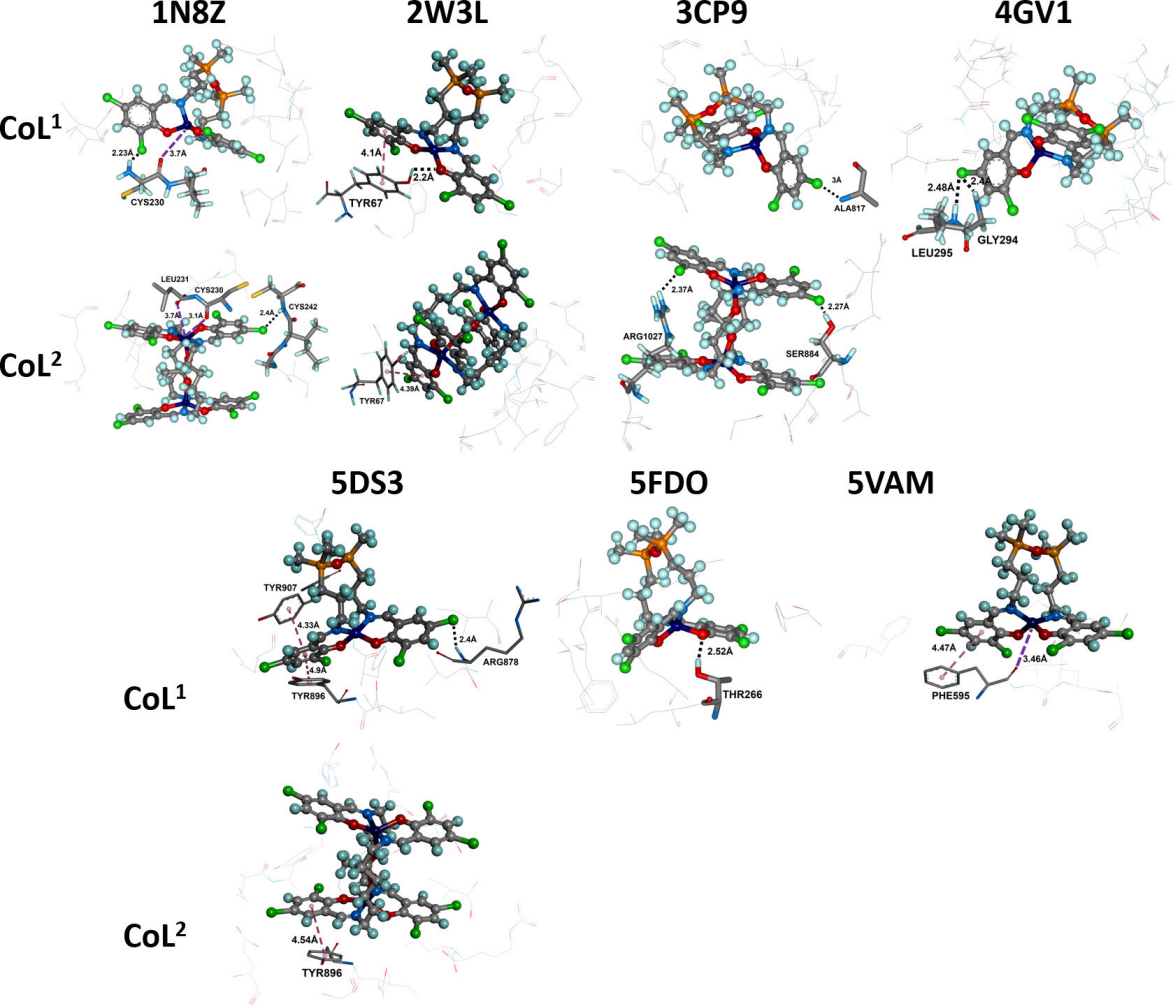
Predicted binding (in line format) of **CoL**^**1**^ and **CoL**^**2**^ with HeLa amino acids within 5 Å. H-bonds are predicted between the side chains of amino acids (stick format) and one of the atoms in the complex (black dashed line), along with a potential chelating interaction with the central metals (purple dashed line), and π···π interactions (pink dashed line; between centroid-centroid in aromatic rings).

Figure 4 illustrates the predicted binding mode of the complexes ***CoL**^**1**^* and ***CoL**^**2**^*. For these proteins, the binding pockets are large, facilitating the accommodation of complex molecules.

It can be observed that the **CoL^2^** molecule is larger and fits more efficiently into the pockets. Additionally, the overlap with the co-crystallized ligand is greater for **CoL^2^**, except for protein 4GV1, for which docking could not be carried out. Similar results were obtained for **H_2_L^1^** and **H_2_L^2^**, as shown in electronic supplementary material, figure S7. For 1N8Z, an interaction was predicted between the metal centre of **CoL^1^** and an oxygen atom from the amino acid CYS230, with a distance of 3.7 Å. The same amino acid forms a hydrogen bridge (NH···Cl) with a chlorine atom from **CoL^1^** at a distance of 2.23 Å. For **CoL^2^**, two interactions were predicted between the metal centre and oxygen atoms from LEU231 and CYS230, with distances of 3.7 and 3.1 Å, respectively.

A hydrogen bond (NH···Cl) between CYS242 and a chlorine atom from **CoL^2^** was also predicted (figure 4). For 2W3L, a π···π interaction was predicted between the aromatic ring of TYR67 and one of the aromatic rings in **CoL^1^** (4.1 Å) as well as **CoL^2^** (4.39 Å). Apart from **CoL^2^**, in **CoL^1^** a hydrogen bond (OH···O, 2.2 Å) between the phenolic oxygen atom of TYR67 and an oxygen atom from the complex was also predicted. For 2CP9, a hydrogen bond (NH···Cl) between ALA817 and a chlorine atom from **CoL^1^** is possible.

Two stronger hydrogen bridges were predicted for **CoL^2^**, compared with the case of **CoL^1^**. Hydrogen bonds are established between ARG1027 and a chlorine atom (NH···Cl), with a distance of 2.37 Å, and between SER884 and chlorine (OH···Cl), with a distance of 2.27 Å. **CoL^1^** forms two hydrogen bridges (NH···Cl) with LEU295 and GLY294 from 4GV1. For 4HJO, no significant interactions between **CoL^1^**/**CoL^2^** and the protein were identified. In the case of 5DS3, it was predicted that **CoL^1^** establishes weak π···π interactions with TYR896 and TYR907, with distances of 4.9 and 4.33 Å. A hydrogen bond (NH···Cl) between ARG878 and a chlorine atom of **CoL^1^**, located on the aromatic nucleus that is not involved in the π···π interactions, was also predicted. For **CoL^2^**, only a π···π interaction (4.47 Å) between TYR896 and an aromatic ring in the compound was predicted. For 5FDO, OH···O interactions between THR266 and an oxygen atom of **CoL^1^** were predicted, while for 5VAM, a π···π interaction between PHE595 and an aromatic ring of **CoL^1^**, as well as an interaction between an oxygen atom from PHE595 and the metal centre, were predicted. For **CoL^2^**, no significant interactions were evident in either case.

**Figure 5.**
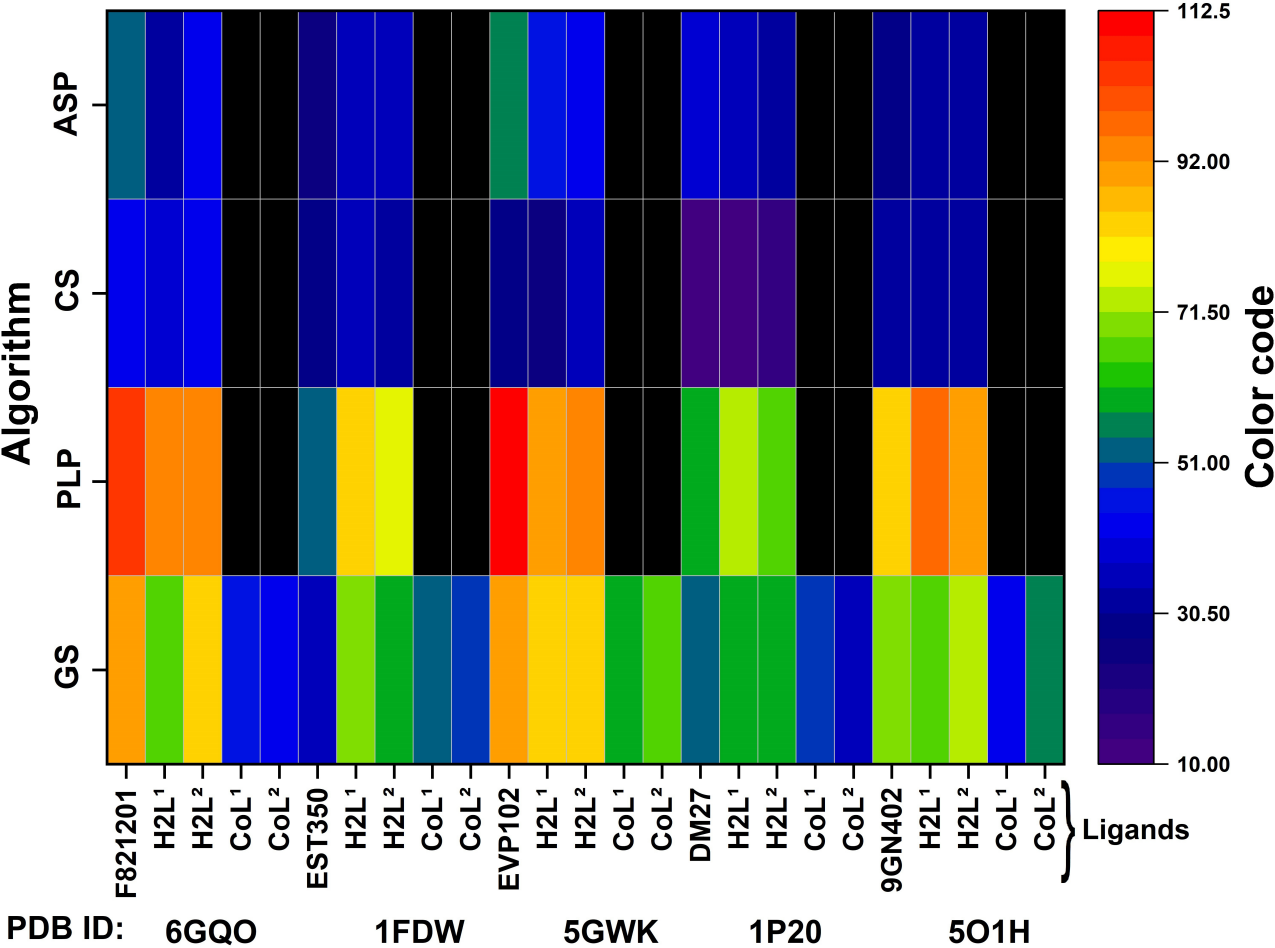
Heatmap representation of predicted binding affinities (depicted using a colour code) for specific proteins (PDB ID) in the MCF-7 cell line with different ligands, using the GS, PLP, CS and ASP algorithms.

For the MCF-7 cell line, based on the literature [68–70], the following structures were selected PDB ID: 6GQO (resolution—1.87 Å) [48], 1FDW (resolution—2.70 Å) [49], 5GWK (resolution—3.15 Å) [50], 1P20 (resolution—1.34 Å) [49], 5O1H (resolution—1.32 Å) [50]. The binding scores in the pockets of this protein of the compounds considered are given in electronic supplementary material, table S2 and graphically represented in figure 5. For **H_2_L^1^** and **H_2_L^2^**, the calculations indicate good docking, as expected, with a more uniform density of interactions for **H_2_L^1^**. This can be attributed to the molecule’s greater flexibility, allowing it to adopt more spatial conformations (electronic supplementary material, figures S10). Given the size of the binding pockets, the molecules can easily penetrate, which explains the high binding scores, in some cases even higher than those of the co-ligand. The calculations suggest that these molecules may exhibit better activity than the related metal complexes.

The complexes show good scores, indicating reasonable binding, similar to the co-crystalized ligand. Only GS runs for the metal complexes.

**Figure 6.**
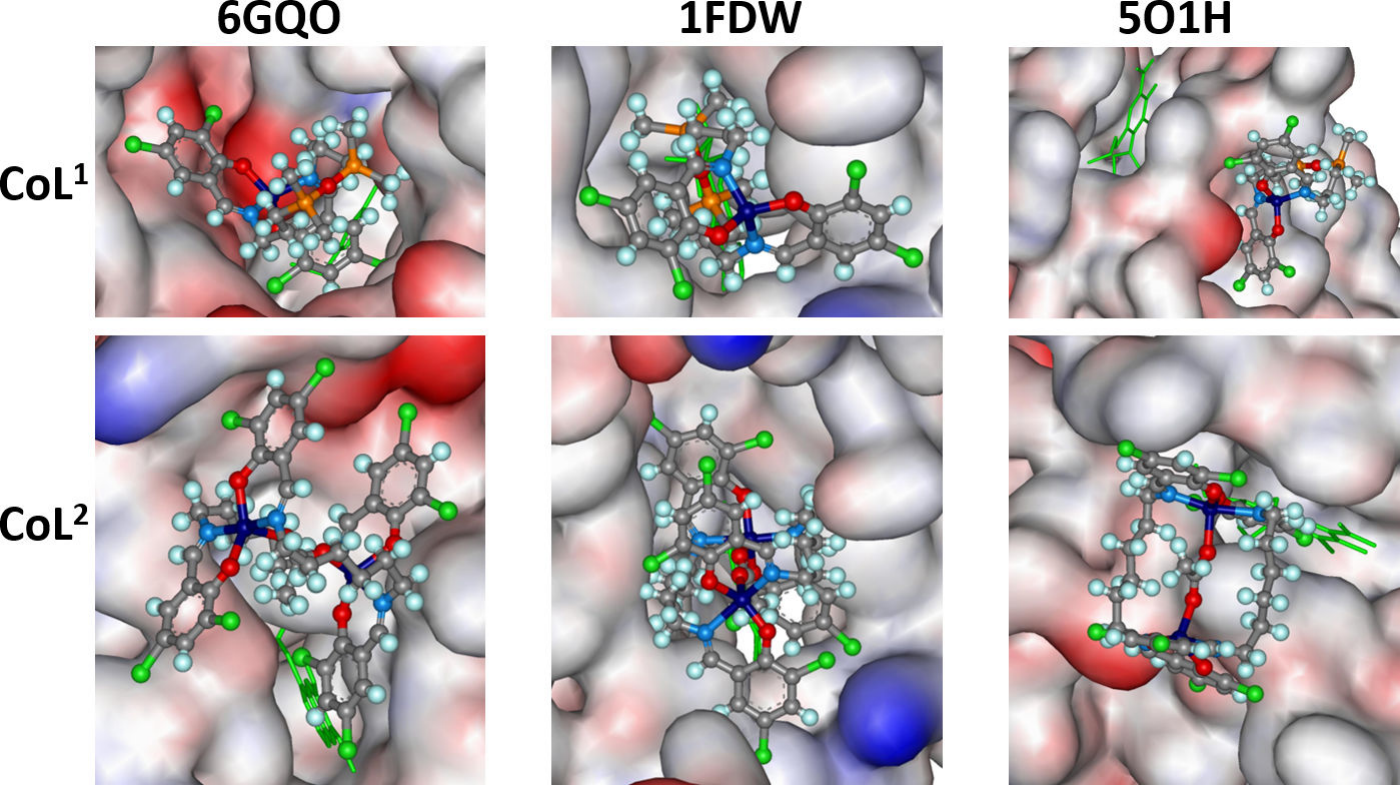
The docked poses of **CoL**^**1**^ and **CoL**^**2**^ (ball-and-stick) in different protein binding sites. The co-crystalized ligand is shown in line format (green), with hydrogens omitted for clarity. The protein surface is rendered, with blue indicating regions with a partial positive charge, red representing regions with a partial negative charge and grey showing neutral areas.

For the studied proteins, the pockets where the co-ligands are located are smaller compared with the previous case, favouring the access of smaller molecules. Molecular docking calculations showed good accommodation of **CoL^1^** within the pockets, while **CoL^2^** was only partially located inside the pocket, with part of the molecule remaining in the aqueous phase (figure 6).

**Figure 7.**
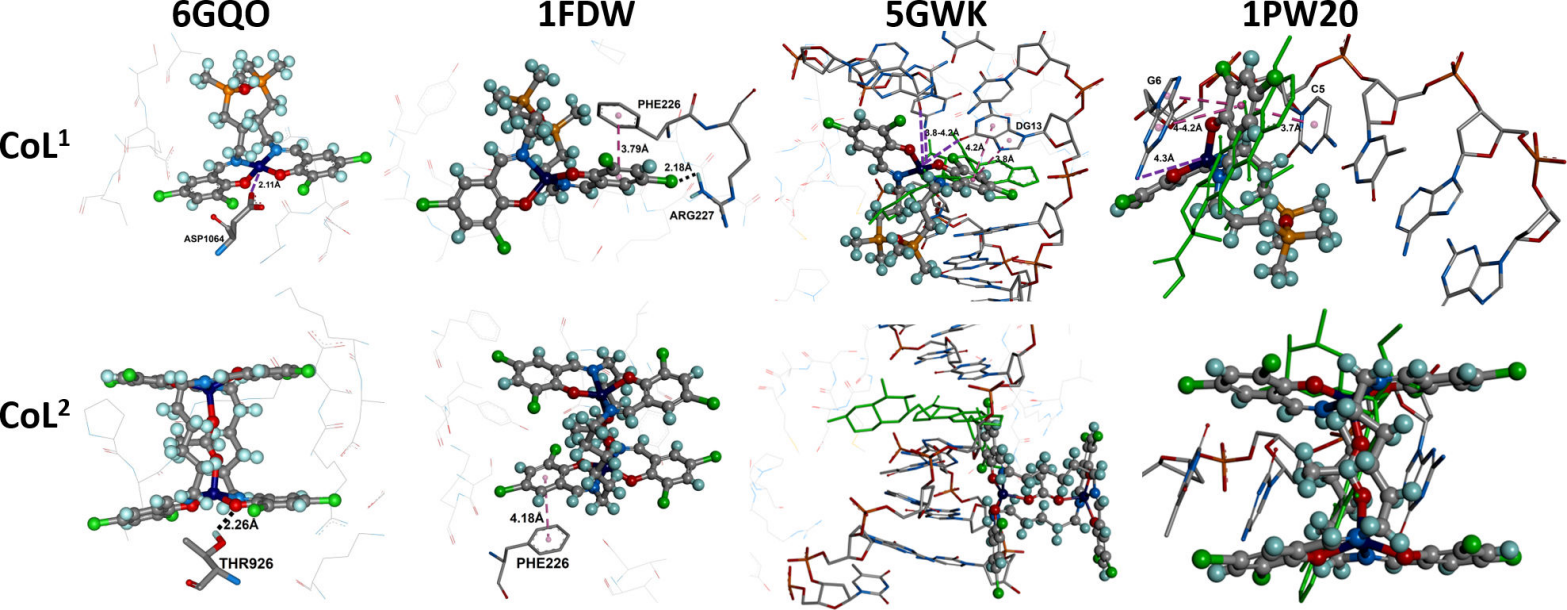
Predicted binding (inline format) of **CoL**^**1**^ and **CoL**^**2**^ with HeLa amino acids within 5 Å. Hydrogen bonding is indicated between the side chains of amino acids (stick format) and one of the atoms in the complex (black dashed line) and a potential chelating interaction with the central metals (purple dashed line) and π···π interactions (pink dashed line; between centroid-centroid in aromatic rings).

In the case of 6GQO, the interaction between **CoL^1^** and the protein consists of a 2.11 Å contact between the metal centre and an oxygen atom from the carboxyl group of ASP1064. For **CoL^2^,** only a hydrogen bond between an oxygen atom and the OH group of THR926 was predicted, with a distance of 2.26 Å. For 1FDW, **CoL^1^** was predicted to exhibit a π···π interaction with a distance of 3.79 Å between its aromatic ring and that of PHE226, as well as a hydrogen bridge with a distance of 2.18 Å between a chlorine atom of the same aromatic ring (involved in the π···π interaction) and a hydrogen atom from the NH_2_ group in ARG277. For **CoL^2^**, only one π···π interaction was predicted with a distance of 4.18 Å between its aromatic ring and the aromatic ring of PHE226 (figure 7). For 5O1H, no significant interactions were identified in either case.

For topoisomerase II (5GWK), calculations indicate that **CoL^1^** is able to interact with the DNA fragment, showing good overlap with co-ligand. Predicted π···π interactions between the aromatic ring and DG13 in the DNA strand were observed, with distances of 3.8 and 4.2 Å. Three interactions with the metal centre were predicted, with distances ranging from 3.8 to 4.2 Å. These summed interactions suggest that **CoL^1^** may be an effective inhibitor of topoisomerase II. Molecular docking calculations for **CoL^2^** indicate that the compound cannot interact with the DNA fragment, which is located outside the strand and helix.

No significant interactions could be identified between the protein and **CoL^2^**. To gain better visualization, the compounds were docked into a DNA fragment (1P20). The calculations predict that **CoL^1^** can interact with the DNA fragment, forming π···π interactions with distances between 3.7 and 4.2 Å, as well as an interaction between the metal centre and a nitrogen atom of G6 at a distance of 4.3 Å. For **CoL^2^**, the results reveal that the compound cannot interact with the DNA fragment, and no significant interactions with the DNA strand were identified.

### 3.3. Biological activity assessment

Siloxanes and hydrocarbons are known as lipophilic moieties. This valuable property can ensure a better interaction of the molecules containing them with the double lipid bilayer of the living cells [17]. Lipophilicity is often correlated with absorption, distribution, metabolism and excretion (ADME) behaviour of drugs and defines their pharmacokinetics and pharmacodynamics. This property provides information related to intra- and inter-molecular forces responsible for drug transport through lipid layers of the cell membranes. While the hydrophobicity refers to the tendency of non-polar molecules to associate in aqueous media, lipophilicity is a physico-chemical property defined as a balance between two categories of forces: hydrophobic/dispersive forces (hydrophobicity) and electrostatic/hydrogen bonds (polarity). Lipophilicity is involved in the solubility, reactivity and degradation of drugs. This property is described by partition coefficient (cLogP) (table 1) meaning the ratio of the concentration of a compound in organic (usually octanol) and aqueous medium under equilibrium conditions.

**Table 1.** The drug-like properties of Schiff bases and cobalt complexes.

compound	cLlogP^a^	M_w_ g mol^−1^	H-bond donors	H-bond acceptors	dipole moment (Debye)^b^
H_2_L^1^	9.84	592.07	<5	<10	1.01
H_2_L^2^	7.64	460.02	<5	<10	5.45
CoL^1^	11.25	648.98	<5	<10	7.00
CoL^2^	18.04	1092.83	<5	<10	11.19

^a^ Calculated using the tools from the ChemDraw16 software package.

^b^ Calculated using the Gaussian16 program, employing the B3LYP method and the 6−311g(d,p) basis set.

Based on this value, the toxicity of a compound can be estimated. According to Lipinski’s ‘rule of five’, the partition coefficient should be less than 5 to suggest drug-likeness potential, while values above 5 indicate that a drug candidate may have poor absorption and permeability. However, the rule of five also takes into account the molecular mass, M_w_, between 400 and 600 g mol^−1^, the number of hydrogen bond donors less than 5 and the number of hydrogen bond acceptors less than 10 [71].

Lipinski’s rule applied to the studied Schiff bases and cobalt complexes revealed higher values of cLogP, suggesting more hydrophobic behaviour of the siloxane ligand **H_2_L^1^** and its complex. Moreover, it is observed an increase in lipophilicity of the ligands by complexation of cobalt ions, especially for **CoL^2^**. This behaviour leads to low solubility in water and decreased permeability through the membrane. M_w_ also deviates to a greater (metal complexes) or lesser (Schiff base ligands) extent of this rule. However, the permeability is also dependent on dipole moment. The calculated dipole moment values revealed amphiphilic compounds, which can interact with cell membranes by electrostatic interactions. Despite deviations from Lipinski’s rule, all compounds revealed a potential permeability across the membrane. Lipinski’s rules have been employed to predict the biological activity of the compounds, but the applicability of these rules to date also demonstrated the existence of some limitations. Lipinski also stated that these parameters must be considered as filters in the selection of the drugs and their interpretation must be considered in the context as each molecule exhibits hydrophilic or hydrophobic groups involved in the modulation of physico-chemical behaviour. Such deviations have been reported for a series of macrocycles and peptides with high molecular weight but with a considerable bioavailability due to increased lipophilicity. The extrapolation of these rules was also found in prodrugs (‘drug latentation’) with attached lipophilic moieties that can further be cleaved promoting the ‘active’ drug [72]. Lipinski’s rule cannot fully predict the pharmacological behaviour of the molecules, so a preliminary screening on normal and cancer cells was assessed.

**Figure 8.**
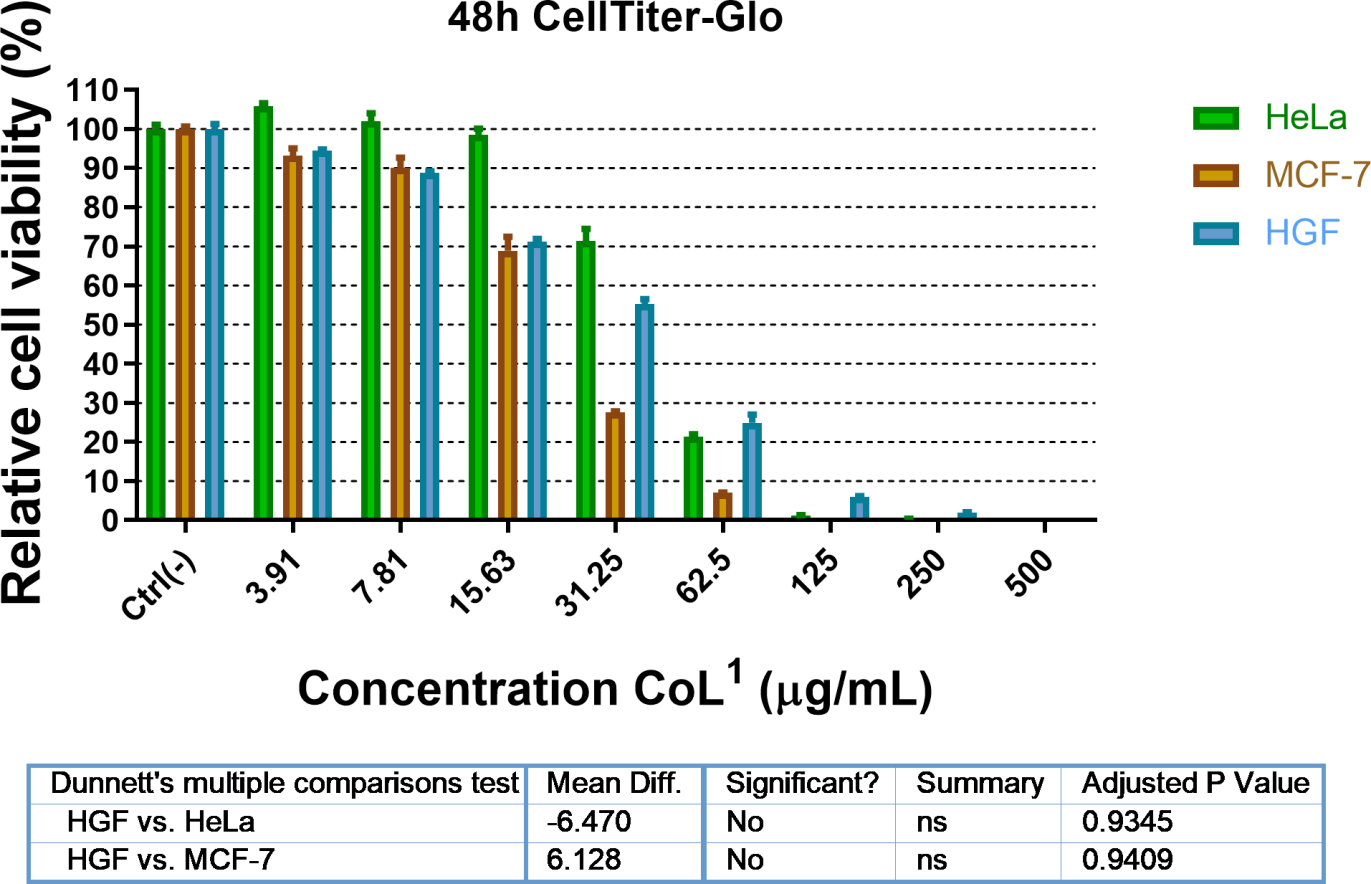
The effect of **CoL**^**1**^ on studied cell lines as a function of concentration. The differences in effect between the three cell lines are not statistically significant, as a result of the ANOVA test.

**Figure 9.**
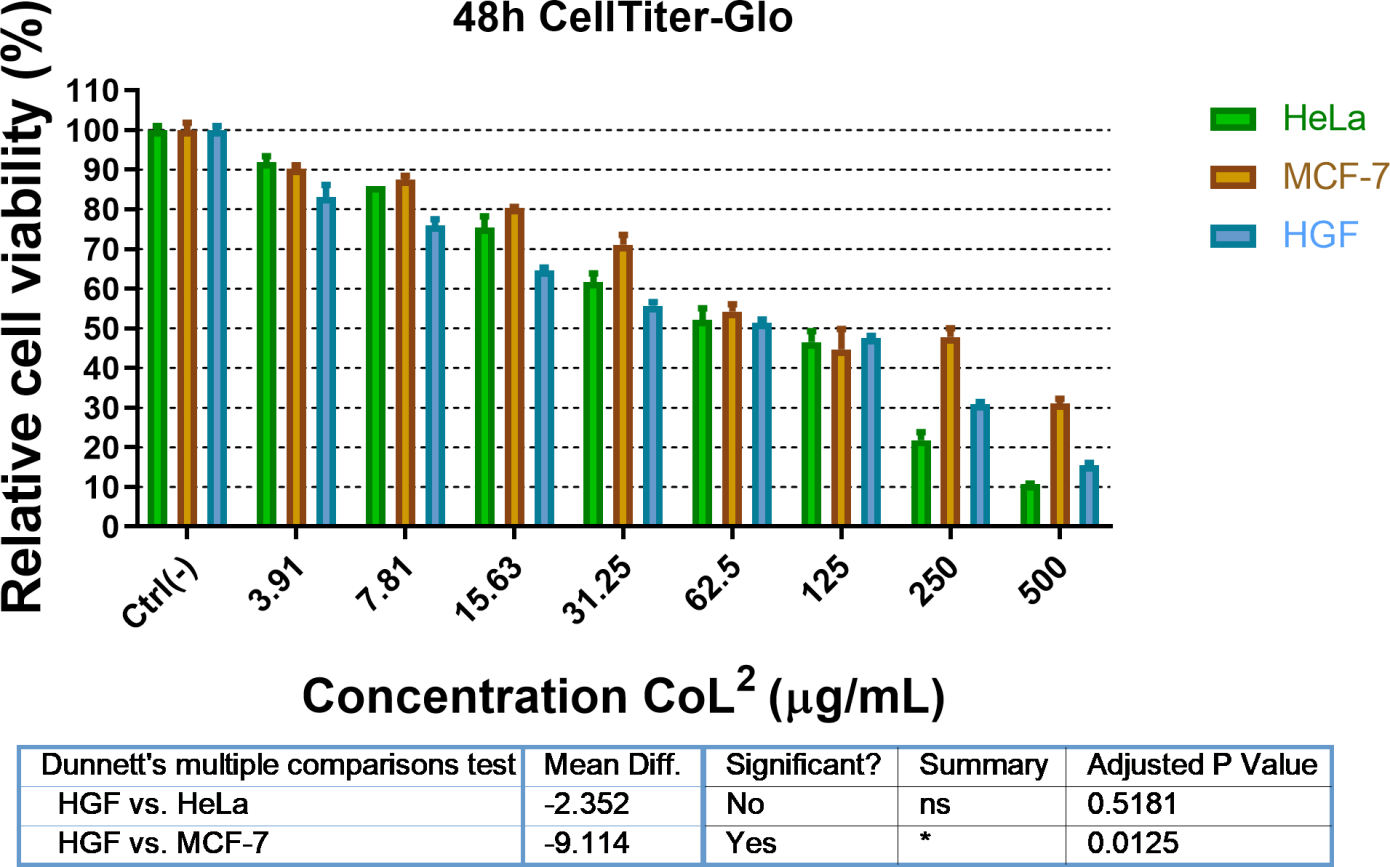
The effect of **CoL**^**2**^ on studied cell lines, as a function of concentration. There are statistically significant differences between HGF and MCF-7 (*p* = 0.0125).

Within the Co(II) complexes, we aimed to see which of the aforementioned (siloxane and alkyl) moieties ensures biocompatibility and strikes better against cancer cells. The anti-tumour activity was investigated by measuring viability of normal cells (human gingival fibroblast, HGF) and two cancerous cell lines (MCF-7 and HeLa) treated with **CoL^1^** and **CoL^2^** in a range of concentrations (3.9–500 μg ml^−1^) over a period of 48 h (figures 8 and 9). Cisplatin was used as standard compound and tested on HGF and MCF-7 cell lines in a lower concentrations range of 0.09–100 μg ml^−1^ (electronic supplementary material, figure S11). The CellTiter-Glo assay is a very sensitive method based on chemiluminescence that generates a light signal that depends on the amount of adenosine triphosphate (ATP) present. Because the amount of ATP is proportional to the amount of living cells, CellTiter-Glo is a very sensitive method for assessing cell viability.

**Figure 10.**
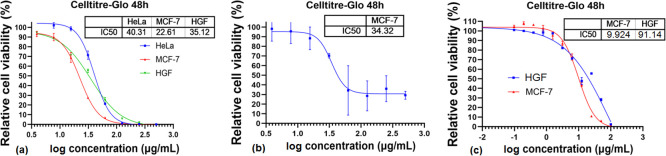
The relative cell viability as IC_50_ of **CoL**^**1**^ (a), **CoL**^**2**^ (b) and cisplatin (c).

As shown in figures 8 and 9, the complex **CoL^1^** has a stronger inhibition effect on MCF-7 cell line than **CoL^2^** at lower concentration (15.63 μg ml^−1^), reducing the cell viability to 70%. The same concentration of **CoL^2^** produces a similar effect only to HGF and HeLa cell lines, indicating a lack of selectivity of this complex, even at twofold concentration (31.25 μg ml^−1^). However, at this concentration (31.25 μg ml^−1^) both complexes exhibited a cytotoxic effect on HeLa cells. The inhibition effect of **CoL^1^** was noticed for MCF-7 cells, showing a high selectivity at this concentration as compared with HeLa cells. The same effect was reached by **CoL^2^** at concentrations fourfold higher on the same cell line. The results of the assessed cytotoxicity were better analysed by means of cell inhibition concentration expressed as IC_50_ values. The IC_50_ of the **CoL^1^** for HeLa, MCF-7 and HGF are 40.31, 22.61 and 35.12 μM, respectively, while in the case of **CoL^2^**, IC_50_ for MCF-7 was 34.32 μM, indicating that **CoL^1^** is more efficient in affecting the cells viability (figure 10a,b). Better solubility and lyophilicity, higher flexibility and lower molecular weight could be the basis for the higher biological activity of the complex containing the siloxane segment.

For complex **CoL^2^** there is no concentration-dependent reduction of the cell viability for HGF and HeLa, more effective being for MCF-7. The viability on normal and MCF-7 cells was compared with that of cisplatin (the common anti-cancer agent), and the results (figure 10c) revealed an IC_50_ for HGF and MCF-7 of 91.14 and 9.924 μM, respectively, indicating a great selectivity, SI (SI = the ratio between the IC_50_ of normal cells and the IC_50_ of MCF-7 cell line), of cisplatin (SI = 9.18) toward MCF-7. Despite the slight reduction of MCF-7 cell viability (2.3-fold smaller than cisplatin), complex **CoL^1^** exhibited a SI of 1.55, much higher than on HeLa cell line. It is true that a lower SI indicates increased off-target effects and narrowing of the therapeutic window, requiring dose reduction below the maximum tolerated dose to minimize toxicity. There are several strategies to improve the selectivity of therapeutic compounds, such as structural modifications of the compounds (molecular redesign and optimization of shape complementarity and electrostatic interactions with the target), combination therapy (use of synergistic drugs with different mechanisms) targeted delivery (conjugation of compounds with antibodies or specific ligands to direct them to diseased cells). However, the *in vitro* cytotoxicity of **CoL^1^** and **CoL^2^** on MCF-7 cell line suggested that these complexes have the potential to act as anti-cancer drugs, taking into account that the efficacy of cisplatin is often reduced in multidrug-resistant cancer cells.

A low systemic toxicity of cobalt complexes was already reported, with a dose-dependent cell death from 76 to 165 mg kg^−1^ [73]. While cisplatin complex induces toxicity by binding DNA (distorsion of the second structure of DNA and blocking DNA transcription and replication) promoting apoptosis, cobalt complexes show a reactivity-dependent oxidation state toxicity. SAR (structure-activity relationship) needs to be elucidated to find the pharmacological potential of cobalt Schiff base complexes. Cobalt(III) complexes may appear inert (pro-drugs) and can be activated in reducing medium, as hypoxia generated by cancer cells in solid tumours, undergoing a bioreductive activation. A cobalt(II) complex of Schiff base containing *p*-aminophenyl-morpholine units has demonstrated a reduced effect on hepatocellular carcinoma cells with IC_50_ in millimolar range. IC_50_ values in the range of 45−100 μM were reported for Co(II) complexes of 2,6-diethyl-phenyl-iminomethyl-pyridine on HeLa cells. The Co(III) complex derived from ethylene diamine and salicilaldehyde exhibited a low toxicity on MCF-7 cancer cells at an IC_50_ < 100 μM [74–76]. A similar cytotoxic effect to our compounds was reported for: cobalt complexes with tris(bipyridine) ligands, where IC_50_ values range from 2.79 to 37.2 μM on different cancer cells, while for normal cells IC_50_ was 13.2 μM, demonstrating a slight selectivity as compared with our complexes [77]; mononuclear Co(II) complex of Schiff base derived from 5-chloromethylisophthaldehyde and phenylpropanolamine (PPA), where IC_50_ = 23.19 μM was found on MCF-7 cell line [78].

## 4. Conclusion

Two cobalt complexes, **CoL^1^** and **CoL^2^,** with salen-type Schiff bases, differing by the ligand bridge, bis(propyl)tetramethyldisiloxane (**H_2_L^1^**) and hexamethylene (**H_2_L^2^**), respectively, were investigated both theoretically and experimentally for their anti-cancer activity. Molecular docking revealed that the unique conformational flexibility and lipophilicity imparted by the silicone moiety in the **H_2_L^1^** ligand and the **CoL^1^** complex facilitate better binding through hydrogen bonding and π···π stacking interactions, leading to enhanced stabilization within protein-binding sites and DNA intercalation compared with **H_2_L^2^** and **CoL^2^**. Despite the deviations from Lipinski’s rule, the amphiphilic nature and dipole moments of these compounds suggest that they can still penetrate cell membranes efficiently, as supported by preliminary cellular assays. In experimental cytotoxicity tests, **CoL^1^** exhibited stronger and more selective inhibition of MCF-7 cells at lower concentrations, with IC_50_ values indicating higher potency compared with **CoL^2^**.

The observed efficacy of **CoL^1^** demonstrated significant anti-tumour activity, even in comparison with cisplatin, although the selectivity index was more favourable for the latter. These findings suggest that the integration of silicon-based motifs in metal-based anti-cancer agents may enhance therapeutic outcomes. However, the study’s limitations include the lack of *in vivo* validation and limited structural diversity of the tested complexes. Future research should focus on evaluating the safety and efficacy of siloxane-modified Co(II)-salen complexes in biological systems, as well as conducting SAR studies to optimize their pharmacological properties. Structural modifications of the compounds that could exploit differences between target and off-target binding sites could also be addressed in future studies.

## Data Availability

Supplementary material is available online [79].
